# Tailored Media Are Key to Unlocking the Diversity of Endophytic Bacteria in Distinct Compartments of Germinating Seeds

**DOI:** 10.1128/spectrum.00172-22

**Published:** 2022-07-18

**Authors:** Davide Gerna, David Clara, Dorothee Allwardt, Birgit Mitter, Thomas Roach

**Affiliations:** a Department of Botany and Center for Molecular Biosciences Innsbruck, University of Innsbruck, Innsbruck, Austria; b Bioresources Unit, Austrian Institute of Technology GmbH, Tulln, Austria; University of Massachusetts—Amherst

**Keywords:** biodiversity, cotyledons, embryonic axis, endophytes, germination, *gyrB*, holobionts, microbiota, parental environment, 16S rRNA, soybean, trophic niche

## Abstract

Seeds offer an internal microbial niche, termed the endosphere, colonized by communities of endophytic bacteria. To elucidate the functions of seed endophytes during germination and early plant growth, studies with culturable isolates are essential. Conventional growth media favor few fast-growing taxa, while micro organisms with restricted nutrient requirements are usually outcompeted prior to isolation. Consequently, current knowledge of the interaction between seeds and their endophytes remains limited to only few bacterial taxa, despite a “black box” of unculturable isolates colonizing the endosphere. Here, we designed various solid media to mimic the endosphere of germinating soybean (Glycine max L.) seeds and assessed their effect on the diversity of culturable endophytic bacteria. The embryonic axis (i.e., the future plant) possessed higher richness and harbored more unique genera (i.e., *Brevundimonas*, *Methylobacterium*, *Microbacterium*, *Pseudoclavibacter*, and *Rathayibacter*) than cotyledons (i.e., seed storage organs). Overall, media containing germinating and ground seeds enabled culturing and isolation of the broadest diversity of endophytic bacteria, viewed through the molecular identification of 246 isolates. The use of multiple tailored media helped uncover trophic adaptation of the core taxa. Furthermore, comparison of seeds from four lots of distinct cultivars and origin revealed few overlapping taxa, indicating that the parental environment, including soil and fertilization regime, influenced seed endophytic diversity. Extended diversity of native seed endophytic bacteria revealed the functional relevance of unique *Arthrobacter*, *Bacillus*, and *Curtobacterium* strains to seed germination under salt stress, exemplifying the importance of enhanced culturing approaches to elucidate the role of microbiota in seed germination.

**IMPORTANCE** Plant growth-promoting endophytic isolates that appear to advance seed germination are often obtained from plant niches other than the seed endosphere. Isolating pure cultures of native endophytes from seeds during germination is crucial to investigate their function during early plant growth. Here, the diversity of endophytic bacteria isolated from seeds during soybean germination was enhanced by combining media tailored to the nutritional composition of the seed endosphere, including pregerminated seeds themselves. Our results show that isolation from distinct soybean seed compartments affected such diversity, with the embryonic axis harboring more unique taxa while displaying higher endophytic richness. Furthermore, using pools of seeds from separate lots, each corresponding to a certain cultivar and field site, supported isolation of further unique strains that often unveiled substantial effects on germination performance. Such findings are relevant to assist studies on the interactions between seeds and their native endophytic bacteria.

## INTRODUCTION

During their entire life cycle, including reproduction, plants harbor and interact with inherent living microbial communities termed microbiotas (1). In spermatophytes, seeds are the outcome of plant fertilization that leads to development of an embryo, the starting point of the new generation. Therefore, for the adult plant, the seed microbiota can be viewed as an inoculum whose characterization is relevant to understanding processes that drive microbial assemblages in the plant holobionts (2–4). Furthermore, seed quality can be affected by the composition of the seed microbiota, which often includes pathogenic species (5–9). Endophytes are the fraction of seed microbiotas that colonizes, for at least part of its lifetime, the so-called endosphere (i.e., internal structures, such as nonembryonic storage tissues and the embryo) (8–10). This definition of endophytes is entirely based on the microbial habitat and not related to mutualism and pathogenicity, traits that require in-depth functional analyses of the holobiont (11). In any case, the various taxa of the endophytic microbiota appear to share some functional traits that enable plant colonization, such as motility, chemotaxis, secretion of cell wall-degrading enzymes, catabolism of plant-released reactive oxygen species, and evasion of plant defenses (10, 12–14). Furthermore, vertical transmission (i.e., seed-mediated heritability) of endophytes may enable their persistence across plant generations (8, 15). Seed-borne endophytes may have the potential to influence seed germination and seedling growth (9, 16–21). However, current evidence for a role of endophytes in germination is largely based on seed inoculation with so-called “growth-promoting” strains isolated from the rhizosphere or other plant-associated microbial habitats, rather than from the seed endosphere itself.

Most plants produce “orthodox” (i.e., desiccation-tolerant) seeds, which accumulate nutritional reserves in species-specific storage organs (i.e., the endosperm, cotyledons, or perisperm) that support heterotrophic growth of the embryo into a seedling (22). In dicotyledon seeds, the radicle and the shoot apex are connected by the hypocotyl and form the embryonic axis, which anchors two cotyledons. In the latest phases of development, orthodox seeds undergo maturation drying and shut down their metabolism (23–25). Thus, inhabiting the seed endosphere requires toleration of resource fluctuations (including water and nutrient availability), which may explain the dynamic phylogenetic changes of endophytic bacteria during seed maturation (8, 9, 26). A restricted number of culturable endophytic bacteria persist during maturation drying, while endospore-forming genera (e.g., *Bacillus*, *Paenibacillus*, and *Curtobacterium*), which are resistant to high osmotic pressure and low water availability, become enriched (16, 27–29). In fully mature and dry orthodox seeds, cells are shrunken, and the cytoplasm forms a glassy matrix with the presence of water limited to local pockets (30, 31), which possibly confine space for the survival of endophytes. During germination (i.e., before radicle protrusion), the endosphere is again substantially modified: seeds rehydrate and increase their fresh weight (FW), while inorganic ions and small organic molecules leak, and seed metabolic activity resumes (22). Protein synthesis reactivates, alongside macromolecular repair and energetic pathways converging to aerobic respiration, which is generally fueled by free amino acids and raffinose family oligosaccharides (32, 33). Furthermore, initial storage reserve mobilization commences, while the embryos and storage tissues interplay with distinct roles in germination metabolism, often displaying species-specific differences (33). Therefore, from the onset of seed germination, water, nutrient, and space availability augment in the endosphere, enabling proliferation of endophytes (8, 12, 16, 34, 35).

Some studies describe the endosphere of a dry seed as a heterogeneous habitat, whereby individual seed compartments (i.e., the embryonic axis and storage tissues) offer distinct niches colonized by certain bacterial taxa (36–39). However, whether trophic niches provided by different seed compartments affect the diversity and abundance of culturable endophytic bacteria during germination remains unknown. Additionally, a role of plant host genotype and cultivar in shaping the diversity of endophytic bacteria through heritability has been suggested (40–42). Nonetheless, influences of plant developmental stage and environmental factors, such as soil, climate, geographical location, and field management practice for crops, are generally acknowledged (8, 17, 26, 43). A recent culture-independent study on the composition of fungal communities of dry seeds showed the significance of parental environmental effects (44). These are defined as external environmental cues and biotic interactions experienced by the parental plant during seed development and maturation drying, with downstream effects on seed vigor and quality (45, 46).

There is consensus that improved understanding of the physiology, taxonomy, ecology, and host interactions of endophytic bacteria cannot disregard culture-dependent approaches. However, many media commonly used to isolate endophytes were originally developed for human-pathogenic Firmicutes and Proteobacteria and are often complex and rich (47). Culturing endophytes on such media may lead to a bias toward copiotrophic taxa with high growth rates that outcompete oligotrophs, which are typically adapted to low nutrient availability. Therefore, minimal media and longer incubation periods may be more suitable for isolating oligotrophic taxa (48–51). Refinement of traditional culturing methods includes medium optimization to better recreate the natural microbial habitat and diversification of *in vitro* growing conditions (48, 52). Optimization through incorporation of plant extracts into both complex rich and minimal media has proved successful for isolating rare taxa and increasing the culture efficiency of plant microbial isolates (47).

Here, we aimed at providing a comprehensive identification of culturable endophytic bacteria isolated from seeds during germination. We hypothesized that metabolic changes occurring in the embryonic axis and storage organs establish different colonization niches, each distinguished by occurrence and diversity of endophytic bacteria. To test this hypothesis, we chose soybean, a model crop of the Fabaceae that produces nonendospermic orthodox seeds with a seed coat and an endosphere comprising two main compartments: an embryonic axis and two cotyledons. We designed soybean-based solid media to mimic the endosphere of germinating seeds and studied their effects on the diversity and abundance of culturable bacterial endophytes in the two seed compartments. To assess the roles of both plant genotype and parental environment on the microbiotas of germinating seeds, endophytic bacteria were isolated from seed lots of different cultivars grown in separate fields within the same geographical area. Finally, the influence on seed germination of the isolated native endophytic bacteria was characterized. Studies on plant-microbe interactions using individual endophytic strains as inoculants suggest that suboptimal and stressful conditions may help improve colonization efficiency of the endosphere, thereby contributing to better elucidation of strain functional traits (53–55). Therefore, germination assays were conducted under salt stress, an emergent abiotic stress factor for most crops, including soybean (56, 57).

## RESULTS

### Seed-based media enabled the isolation of the widest diversity of seed endophytic taxa compared to standard media.

**FIG 1:**
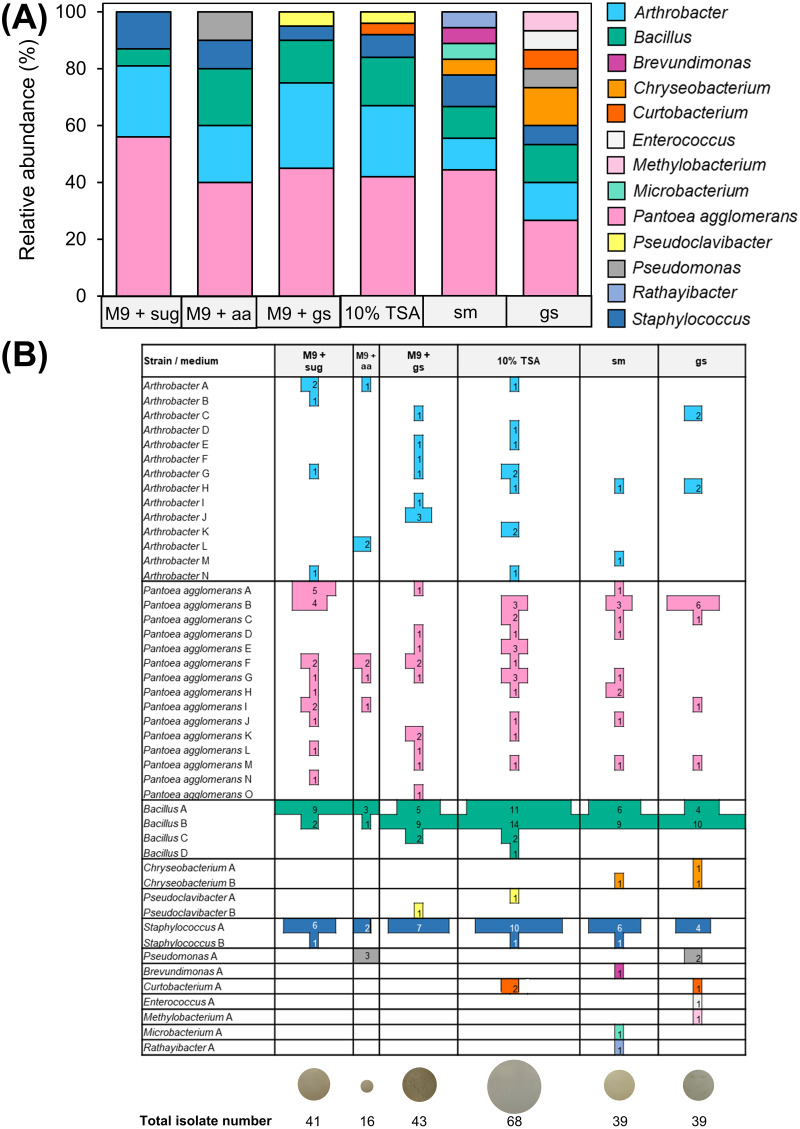
Medium-dependent diversity and richness of culturable bacterial endophytes isolated from soybean seeds during germination. All media were solid (1% [wt/vol] agar) and included, from left to right, M9 minimal salts medium supplemented with either 21 μM glucose and 26 μM galactose (M9 + sug), 4.1 mM glutamine and 0.8 mM glutamate (M9 + aa), or 1.8 to 3.5% (wt/wt) germinating ground seeds (M9 + gs). Other media were prepared using 10% (wt/wt) TSA, 20% (vol/wt) commercial soy milk (sm), and 1.8 to 3.5% (wt/wt) germinating ground seeds (gs). (A) Relative abundance of seed endophytic taxa across media. (B) Strain distribution across media. Colors refer to taxa according to the key in panel A, with bar width and numbers referring to isolates assigned to a certain strain. Areas at the bottom are proportional to the total number of molecularly identified isolates obtained from each medium.

**FIG 2:**
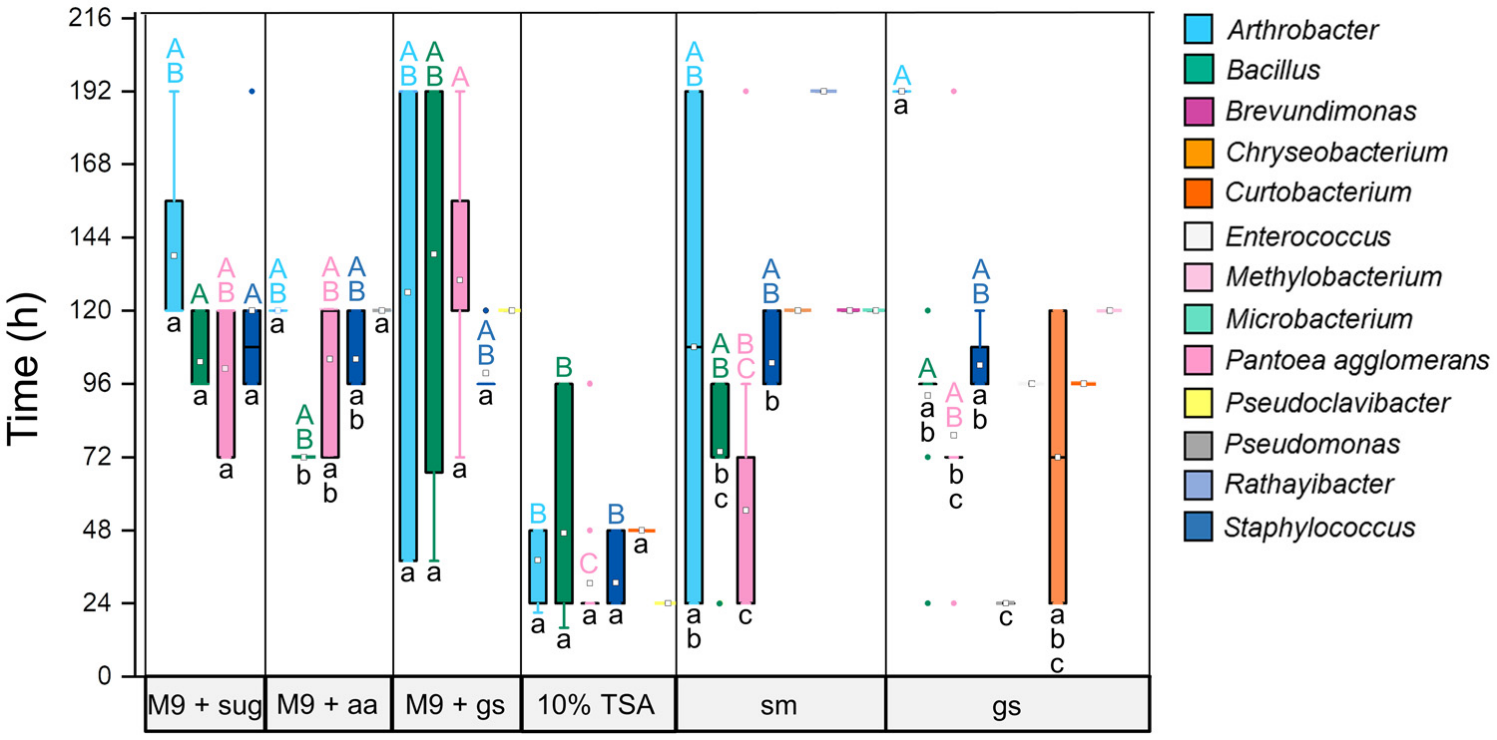
Medium-dependent speed of growth of bacterial endophytic isolates from soybean seeds during germination. The speed of growth at 28°C in the dark was measured as time required visualizing growing colonies ~0.5 to 1 mm in diameter. All media were solid (1% [wt/wt] agar) and included, from left to right, M9 minimal salts medium supplemented with either 21 μM glucose and 26 μM galactose (M9 + sug), 4.1 mM glutamine and 0.8 mM glutamate (M9 + aa), or 1.8 to 3.5% (wt/wt) germinating ground seeds (M9 + gs). Other media were prepared using 10% (wt/wt) tryptic soy agar (TSA), 20% (vol/wt) commercial soy milk (sm), and 1.8 to 3.5% (wt/vol) germinating ground seeds (gs). Each taxon is labeled by a unique color (top right). Open squares indicate average speed of colony growth or single values for isolates of unique genera, small solid circles refer to outliers, and whiskers indicate minima and maxima. Lowercase letters denote differences across taxa cultured and isolated on the same medium, whereas uppercase letters denote differences across the same taxon on various media (nonparametric Kruskal-Wallis tests by ranks followed by the Bonferroni correction for multiple tests; *P < *0.05). Taxa represented by unique isolates were not tested for significance.

First seeds of ‘Abelina’, ‘Amadea’, and ‘Bio-Amandine’ germinated around 63 h after the onset of imbibition, whereas for ‘Cordoba’, first germinations were observed after 50 h (see Fig. S1 in the supplemental material). Therefore, 50 and 40 h were chosen as 80% of incubation times before first germination for isolation of endophytic bacteria from seeds (referred to here as T80 seeds) of the first three cultivars and ‘Cordoba’, respectively. In total, 246 bacterial isolates from T80 seeds were identified through 16S rRNA gene partial sequencing and belonged to the phyla Actinobacteria, Bacteroidetes, Firmicutes, and Proteobacteria (see Table S1 in the supplemental material). The abundant genera *Arthrobacter*, *Bacillus*, and Staphylococcus, together with the species Pantoea agglomerans, were cultured and isolated from all six tested soybean-based media (Fig. 1A). Isolates could be grouped in multiple ribotypes and clusters (referred to collectively here as strains), as revealed by restriction fragment length polymorphism (RFLP) analysis of the 16S-23S ribosomal intergenic spacer (IGS) and DNA gyrase subunit B (*gyrB*) sequence analyses. In particular, 15, 14, 4, and 2 strains were assigned to *P. agglomerans*, *Arthrobacter*, *Bacillus*, and Staphylococcus, respectively (Fig. 1B). About 95% of endophytic bacteria attributed to these genera could be isolated using 10% (wt/wt) tryptic soy agar (TSA), which led to the highest number of bacterial isolates (~28% of the total) across all tested media, and maximal richness, viewed as number of different strains (Fig. 1B). In contrast, the minimal richness was attained using M9 minimal salts medium supplemented with Glu and Gln, whereas each of the remaining media reached intermediate levels of endophytic richness (Fig. 1B). Compared to the other media, the most abundant taxa—*P. agglomerans*, *Arthrobacter*, *Bacillus*, and Staphylococcus—generally grew faster on 10% (wt/wt) TSA, which also enabled isolation of almost the full range of strains assigned to such taxa (Fig. 1 and 2; also, see Fig. S3 in the supplemental material).

Designing media containing T80 ground seeds or soybean milk enabled to culture, isolate, and identify other unique strains, resulting in the largest diversity of endophytic bacteria that included the less frequent genera Pseudomonas, *Brevundimonas*, *Curtobacterium*, *Enterococcus*, *Methylobacterium*, *Microbacterium*, and *Rathayibacter*. Of these, only Pseudomonas and *Curtobacterium* were also isolated from M9 minimal salts medium supplemented with Glu and Gln and with 10% (wt/wt) TSA, respectively. Despite reduced genus diversity (Fig. 1A), the addition of M9 salts to T80 seed-based medium resulted in isolation of some unique strains of the predominant taxa *Arthrobacter*, *Bacillus*, and *P. agglomerans* (Fig. 1B). Strains of each of the less frequent genera *Chryseobacterium* and *Pseudoclavibacter* were obtained mainly after culturing on T80 seed-based media, on which they formed colonies with a speed comparable to that of the more abundant taxa (Fig. 1 and 2). Occasionally, the phenotypes of some isolates grown on unconventional media changed after subculturing. Therefore, to prove purity, 19 isolates were randomly selected and subcultured on both 10% (wt/wt) TSA and 20% (vol/wt) soybean milk. Partial sequencing of the 16S rRNA gene confirmed the identity of the same strains before and after subculturing on both media.

In summary, the standard complex rich soybean-based medium 10% TSA (wt/wt) enhanced the fast growth of a broad range of *P. agglomerans*, *Arthrobacter*, and *Bacillus* seed endophytic strains, but only of few less represented genera. In contrast, culturing endophytes on self-designed seed-containing media, which aimed at mimicking the nutrients of T80 seeds, enabled isolation of unique strains of less abundant genera, such as *Brevundimonas*, *Enterococcus*, *Methylobacterium*, *Microbacterium*, and *Rathayibacter*.

### Embryonic axes hosted a more diverse and richer endophytic bacterial microbiota.

**FIG 3:**
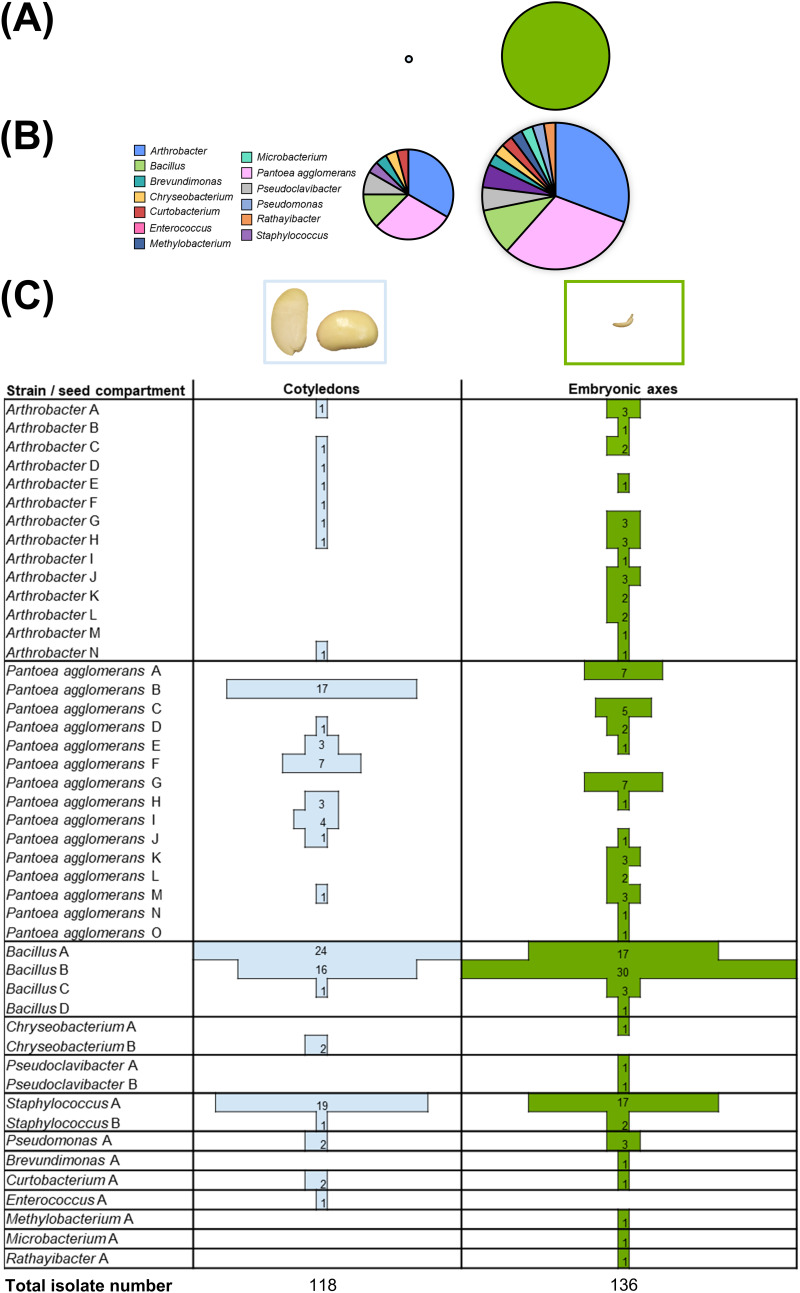
Composition of culturable bacterial endophytes isolated from cotyledons (light blue) and embryonic axes (green) during soybean seed germination. (A) Relative abundance of endophytic strains is expressed by circle area after normalization to the dry weight of each seed compartment. (B) Diversity and richness of endophytes from both seed compartments. Every taxon corresponds to a distinct color. Circle areas and inner sector sizes are proportional to richness (i.e., number of strains) and number of distinct strains within each taxon, respectively. (C) Strain distribution in cotyledons and embryonic axes, with bar width and numbers referring to isolates assigned to a certain strain.

To elucidate possible differences in abundance and diversity of endophytes in distinct seed compartments during germination, embryonic axes were excised from cotyledons prior to isolation of endophytic bacteria. The relative abundance of endophytic isolates obtained from the embryonic axis was about 17-fold larger than that in the cotyledons (Fig. 3A). Furthermore, among all identified taxa, 39 and 25 endophytic strains were isolated from the embryonic axis and cotyledons, respectively, hence leading to a 30% higher richness in the first (Fig. 3B and Table S1 in the supplemental material). *P*. *agglomerans*, followed by *Arthrobacter* and then *Bacillus*, was the richest taxon isolated from both seed compartments (Fig. 3B). However, the embryonic axis harbored a larger number of unique *P. agglomerans* (+53%) and *Arthrobacter* (+50%) strains (Fig. 3C and Table S1 in the supplemental material). In contrast, four and two strains were classified in the genera *Bacillus* and Staphylococcus, respectively, and were in general equally present in both seed compartments (Fig. 3C). Concerning the less abundant genera, the three *Chryseobacterium* isolates were distinguished into unique strains of each seed compartment, while isolates of Pseudomonas and *Curtobacterium* clustered into single strains found in both seed compartments. Finally, *Enterococcus* was isolated only from cotyledons, whereas unique isolates of *Pseudoclavibacter*, *Brevundimonas*, *Methylobacterium*, *Microbacterium*, and *Rathayibacter* were obtained only from embryonic axes (Fig. 3B and C and Table S1 in the supplemental material).

### Plant cultivar and field site influenced the composition of seed endophytic microbiota.

**FIG 4:**
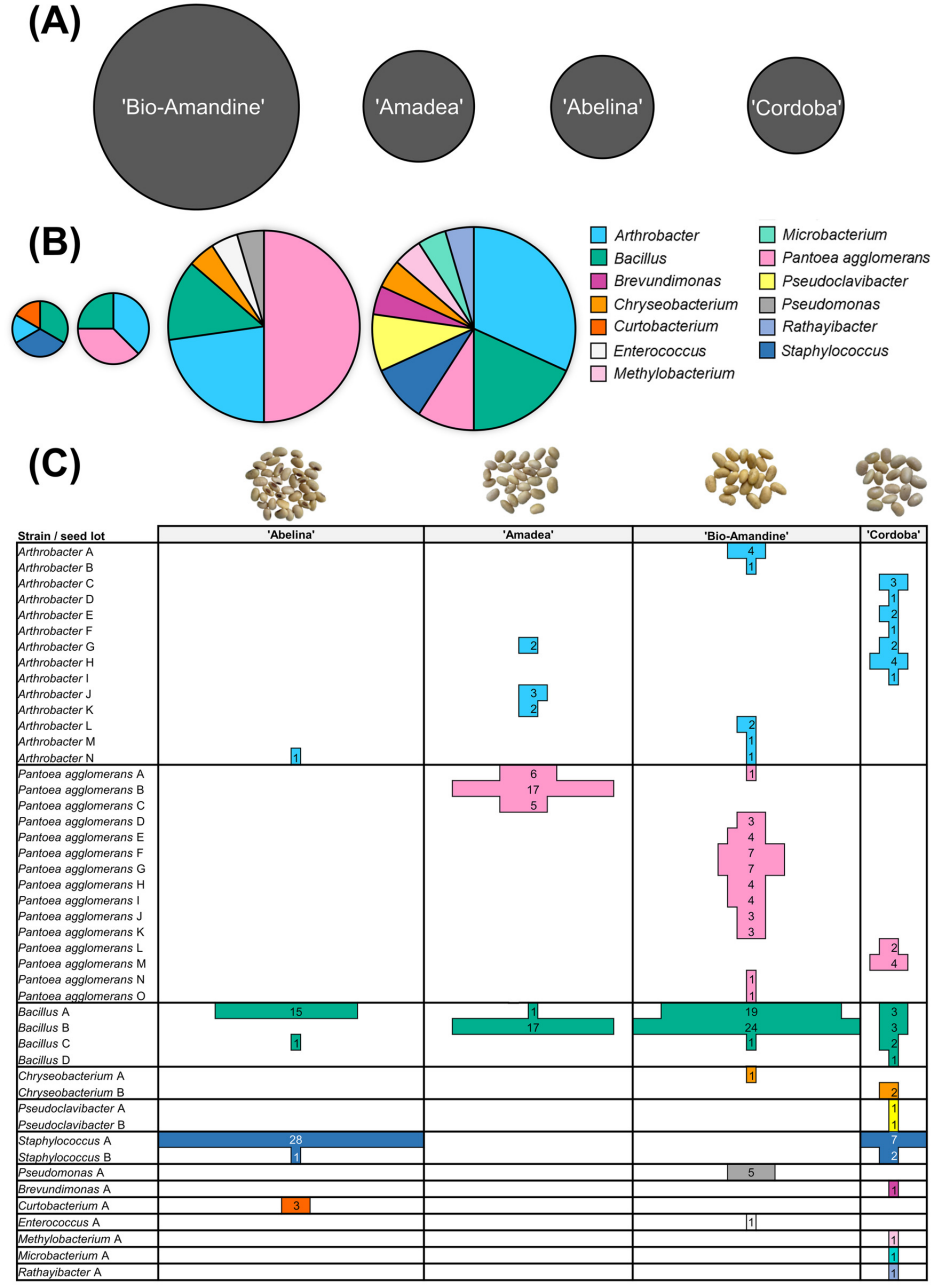
Composition of culturable bacterial endophytes isolated from different seed lots. Each lot consisted of seeds of different soybean cultivars grown in distinct fields (refer to Fig. S2 in the supplemental material). (A) Total numbers of endophytic isolates from each cultivar expressed by circle area. (B) Diversity and richness of endophytes across seed lots of the cultivars ‘Abelina’, ‘Amadea’, ‘Bio-Amandine’, and ‘Cordoba’ (left to right). Each taxon corresponds to a distinct color. Circle areas and inner sector sizes are proportional to endophytic richness (i.e., number of strains) and number of distinct strains within a definite taxon, respectively. (C) Distribution of strains across cultivars, with bar width and numbers referring to isolates assigned to a certain strain.

The isolation of endophytic bacteria from distinct seed lots, each obtained from plants of a certain cultivar and grown in different field sites (Fig. S2 in the supplemental material), clearly affected culturable endophytic communities. In comparison to the other seed lots, a larger number of colonies with heterogeneous phenotypes was obtained after streaking slurries of ‘Bio-Amandine’ seed compartments. As a result, most endophytic isolates (i.e., 40% of the total) originated from the ‘Bio-Amandine’ seeds, whereas the number of isolates from the other seed lots was on average 20% lower (Fig. 4A). The highest number of genera was isolated from seeds of ‘Cordoba’ (ten), followed by ‘Bio-Amandine’ (six), ‘Abelina’ (four), and ‘Amadea’ (three) (Fig. 4B). Similarly, the overall strain diversity, viewed as number of strains uniquely found in one particular seed lot, was increased in ‘Bio-Amandine’ and ‘Cordoba’ in comparison to ‘Abelina’ and ‘Amadea’ (Fig. 4C).

Only two of the 13 identified genera, *Bacillus* and *Arthrobacter*, were isolated from seeds of all lots, with the majority of endophytic isolates assigned to *Bacillus* and with *Arthrobacter* revealing maximal strain diversity (Fig. 4C). Another predominant taxon was *P. agglomerans*, whose isolates were identified in seeds of three seed lots (i.e., ‘Amadea’, ‘Bio-Amandine’, and ‘Cordoba’) as unique strains. Notably, most isolates of the 10 genera with lowest relative abundance (i.e., *Chryseobacterium*, *Pseudoclavibacter*, Staphylococcus, Pseudomonas, *Brevundimonas*, *Curtobacterium*, *Enterococcus*, *Methylobacterium*, *Microbacterium*, and *Rathayibacter*) were identified as seed lot-specific and unique strains, except for *Chryseobacterium* and Staphylococcus, which were both found in ‘Cordoba’ but also in ‘Bio-Amandine’ and ‘Abelina’ seeds, respectively (Fig. 4C).

Altogether, but excluding *Bacillus*, Staphylococcus and Arthrobacter, endophytic strains were isolated from seeds of one lot only (Fig. 4B).

### Impact of medium on the CFU numbers of endophytic bacteria isolated from cotyledons and embryonic axes of four seed lots.

**FIG 5:**
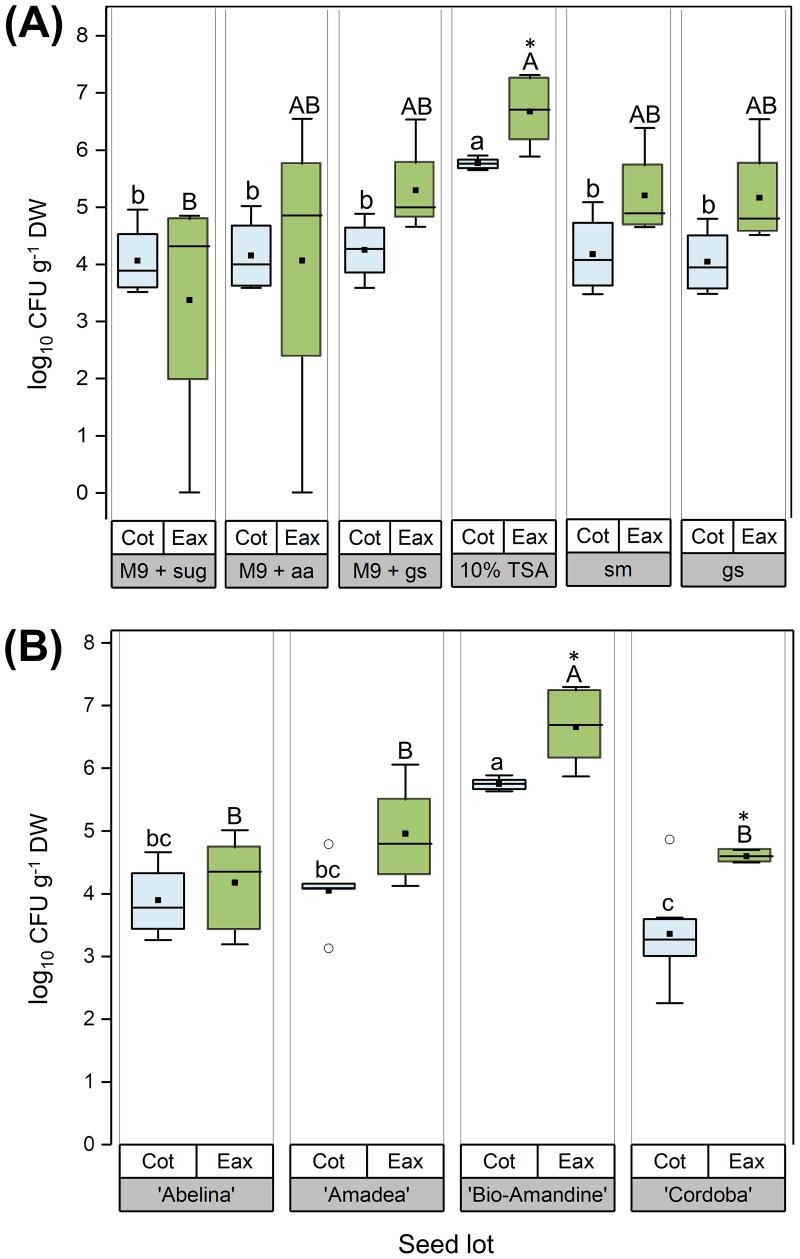
Comparison of CFU numbers of endophytic bacteria isolated from cotyledons (Cot; light blue) and embryonic axis (Eax; green) of soybean seeds during germination. (A) Effects of various solid media on the number of endophytic CFU isolated from a ‘Bio-Amandine’ seed lot. All media were solid (1% [wt/wt] agar) and included, from left to right, M9 minimal salts medium supplemented with either 21 μM glucose and 26 μM galactose (M9 + sug), 4.1 mM glutamine and 0.8 mM glutamate (M9 + aa), or 1.8 to 3.5% (wt/wt) germinating ground seeds (M9 + gs). Other media were prepared using 10% (wt/wt) TSA, 20% (vol/wt) commercial soy milk (sm), and 1.8 to 3.5% (wt/vol) of germinating ground seeds (gs). Closed squares and open circles denote means (4 replicates of 5 cotyledons of different seeds and 10 to 15 embryonic axes each) and outliers, respectively, while whiskers indicate minima and maxima. (B) Effect of 10% (wt/wt) TSA on the number of endophytic CFU isolated from seed lots of ‘Abelina’, ‘Amadea’, ‘Bio-Amandine’, and ‘Cordoba’. Letters denote significant differences across media and seed lots (nonparametric Kruskal-Wallis tests by ranks followed by the Bonferroni correction for multiple tests; *P < *0.05). Asterisks denote significant differences (Mann-Whitney *U* test for independent samples; *P < *0.05) between endophytic CFU numbers of cotyledons and embryonic axes either isolated from the same seed lot (A) or cultured on the same medium (B). The numbers of CFU were normalized over the dry weights (DWs) of each seed compartment.

Previous results were obtained via an approach aimed at targeting the isolation of the broadest endophytic diversity by preferentially selecting colonies with dissimilar phenotypes. However, culturable endophytes in the different seed compartments and seed lots were also assessed quantitatively. First, the influence of the six soybean-based media on the CFU numbers of endophytes isolated from embryonic axes and cotyledons was quantified using ‘Bio-Amandine’ seeds. The majority of endophytic isolates was obtained from this seed lot, which also possessed intermediate relative abundance of taxa and maximal strain diversity (Fig. 4). Across media, the average number of CFU in the embryonic axis was generally higher (~10^5^ g dry weight [DW]^−1^) than in cotyledons (~10^4^ g DW^−1^), but not significantly, except on 10% (wt/wt) TSA. On this medium, the CFU number rose by 1 order of magnitude, and ~14-fold more CFU originated from the embryonic axis than from cotyledons (Fig. 5A). Therefore, 10% (wt/wt) TSA was selected to quantify differences across seed lots. The CFU number of endophytes from cotyledons and embryonic axis together was, on average, 6.4-fold larger in ‘Bio-Amandine’ than in the other seed lots (Fig. 5B). Similar to ‘Bio-Amandine’, counting on 10% (wt/wt) TSA showed that 27-fold more CFU originated from embryonic axes than cotyledons of ‘Cordoba’, while no significant differences were detected in ‘Abelina’ and ‘Amadea’ seeds (Fig. 5B).

### Native endophytic bacteria differentially affected seed viability and speed of germination under salt stress.

**FIG 6:**
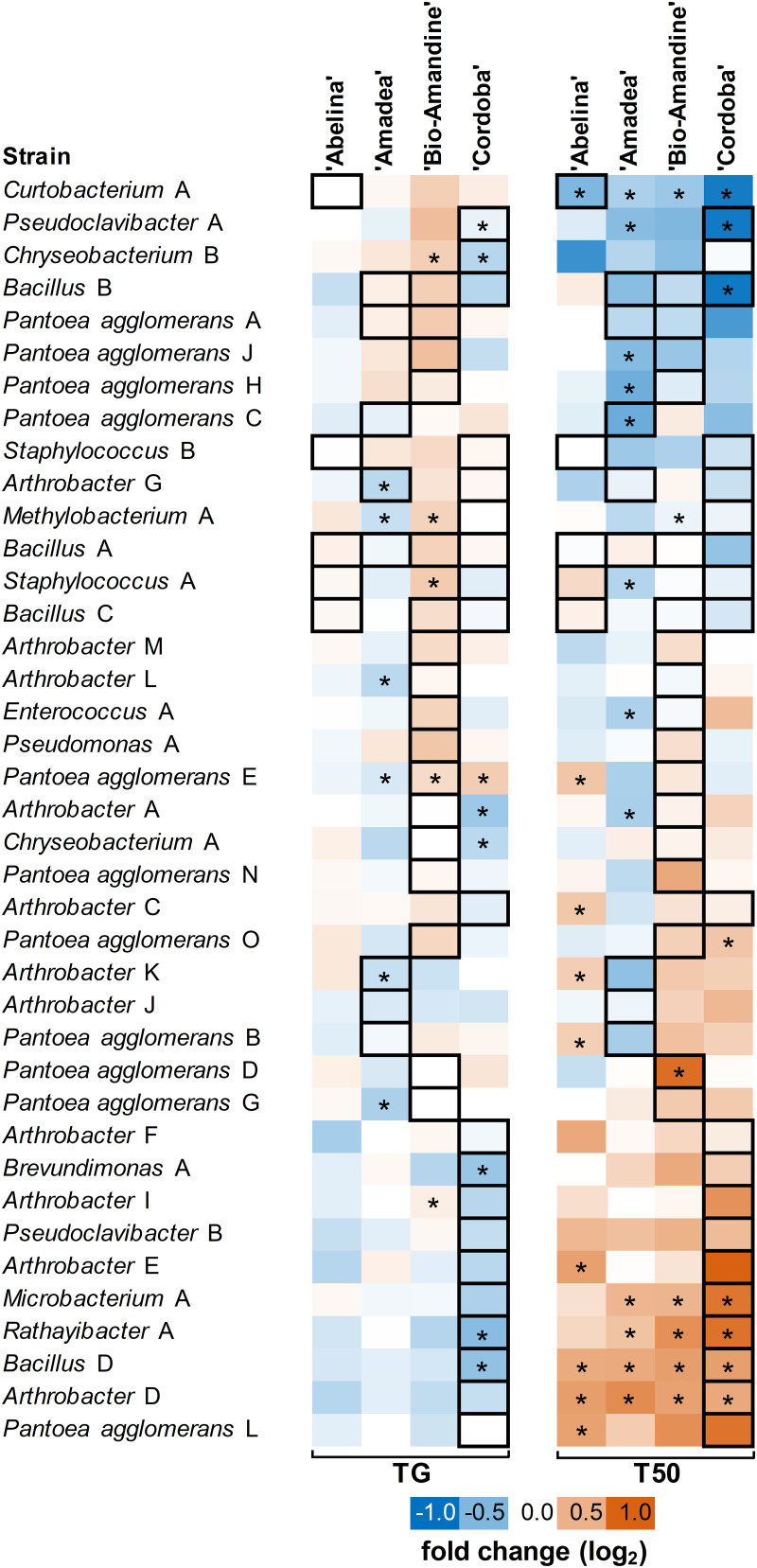
Influence of endophytic bacteria on seed germination of ‘Abelina’, ‘Amadea’, ‘Bio-Amandine’, and ‘Cordoba’ soybean under salt stress. Effects are shown as fold changes in total germination (TG; i.e., viability) after 7 days at 20°C in the dark with 300 mM NaCl, and time to 50% germination (T50; i.e., vigor) of seeds inoculated with liquid suspensions of individual strains relative to control seeds under the same conditions. Blue and orange indicate decreases and increases, respectively, as illustrated by the color scale at the bottom. Thick borders denote the seed lot(s) from which a certain endophytic strain was isolated. Values are means (3 replicates of 15 seeds each), and asterisks indicate significant differences from the controls (Mann-Whitney *U* test for independent samples; *P < *0.05).

All chosen bacterial strains were able to grow on 10% (wt/vol) TSA supplemented with NaCl to a final concentration of 300 mM. Therefore, they could be inoculated individually to test their impact on seed germination performance at 300 mM NaCl. Overall, effects on total germination (i.e., seed viability) under salt stress were modest, whereas the speed of germination was more pronouncedly influenced. Strains displayed slight germination-promoting, pathogenic, and neutral effects, depending on the seed lot (Fig. 6). In fact, seeds of the various lots responded differently to “self”- and “cross”-inoculations (i.e., inoculation with strains either isolated from the same lot or not, respectively). Cross-inoculations of ‘Abelina’ and ‘Bio-Amandine’ seeds with strains from other lots mostly led to significant deceleration of germination (Fig. 6). ‘Amadea’ and ‘Bio-Amandine’ seeds self-inoculated with one strain each germinated significantly faster and slower, respectively. Following self-inoculations, four strains accelerated ‘Cordoba’ seed germination. Altogether, one *Bacillus* and one *Arthrobacter* strain slowed seed germination of all four lots under salt stress, as opposed to a unique *Curtobacterium* strain that significantly sped up germination of all lots (Fig. 6).

## DISCUSSION

### Combining multiple seed endosphere-based media enabled isolation of a broader diversity of seed endophytes.

Both culture-dependent and -independent analyses coupled with molecular identification are fundamental to advancing our understanding of the role played by endophytic bacteria during seed germination. High-throughput genome sequencing has enabled less biased assessments of the microbial diversity, community composition, and dynamics in the endosphere, hence advancing state-of-the-art knowledge on the ecology of endophytes (54, 58, 59). Nonetheless, understanding the physiological role and *in vivo* interactions of seed endophytes requires studies with microbial isolates, whose diversity can be enhanced from developing novel culturing techniques (47, 48, 52, 60, 61). Plant tissues and their extracts provide a broad range of macromolecules, vitamins, and inorganic salts whose use, either as supplements to standard complex media or as unique nutritional sources, can improve culturing of endophytic bacteria and regeneration from glycerol stocks (62, 63). However, mimicking the nutritional composition of the endosphere to determine influences on the diversity of seed endophytes is uncommon.

Here, we targeted *in vitro* culturing of soybean endophytes from germinating seeds and compared their diversity and richness across 10% (wt/wt) TSA, a soybean-based standard complex medium, minimal M9 media tailored to the major monosaccharides and amino acids of seeds during germination, and seed-based media, including T80 seeds and commercial soybean milk. The latter is obtained from soaked or pregerminated seeds (64), and, along with the medium supplemented with T80 seeds only, enabled culturing of the largest diversity of endophytic bacteria (Fig. 1A). Thus, using seed-based media that best simulated the seed endosphere during germination promoted isolation of unique taxa (Fig. 1A and B), supporting findings of similar studies on endophytic bacteria of other plant-associated habitats (61–63, 65–67). In contrast to core taxa, those that occur in certain niches, developmental stages, and genotypes of their plant host are termed “satellite” and, albeit of low abundance, can drive microbial dynamics of the holobiont (68–70). *Brevundimonas*, *Chryseobacterium*, *Enterococcus*, *Methylobacterium*, *Microbacterium*, and *Rathayibacter* appeared as satellite endophytic genera, which were isolated only using media consisting of T80 seeds (Fig. 1), hence suggesting adaptation and specialization to the seed endosphere. In fact, these satellite genera were also not detected on media containing highly concentrated inorganic M9 salts, originally developed for studies of Escherichia coli (71, 72). Furthermore, satellite genera did not form visible colonies earlier than 4 days (Fig. 2 and Table S1 in the supplemental material). Pseudomonas was another rarely isolated genus with few isolates attributed to a unique strain, which showed faster growth in the presence of Glu and Gln as sole carbon sources (Fig. 1 and 2). Considering that such amino acids become predominant during soybean germination (73), they are probably critical nutrients for the occurrence of this Pseudomonas strain in the seed endosphere.

The most abundant seed endophytes, which were assigned to the core taxa *P. agglomerans*, *Bacillus*, and Staphylococcus, displayed broad nutritional versatility but fastest growth and maximal richness on the nutrient-rich 10% (wt/wt) TSA (Fig. 1 and 2), indicative of copiotrophy (51). Enrichment in copiotrophic bacterial taxa arises during seed germination and seedling growth (34, 35), suggesting that efficient carbohydrate and amino acid catabolism could facilitate persistence of these taxa in the seedling endosphere. Versatility and adaptability of copiotrophic taxa to different media were accompanied by different colony morphologies (our unpublished observation), as is typical of some endophytes (68). Furthermore, unique strains of *Arthrobacter* were often isolated from only one of the six media, indicating distinct trophic requirements within this abundant core genus (Fig. 1), perhaps even influencing strain distribution between seed compartments (Fig. 3C).

In summary, our results on soybean show that combining different media tailored to the endosphere of seeds during germination was advantageous for isolating unique bacterial genera and better revealing trophic adaptation of the dominant taxa.

### Heterogeneity of endophytes between compartments of germinating seeds: implications for seed-mediated heritability.

Knowledge of mechanisms of endophyte transmission and heritability via seeds, viewed as a reservoir of taxa for the plant progeny, are relevant for elucidating the dynamics of microbiota assemblage (8). During seed germination, embryonic axes hosted a richer and more diverse bacterial endophytic microbiota than cotyledons (Fig. 3), despite an 88% lower dry mass. When comparing the effect of media, 10% (wt/wt) TSA increased the CFU number of endophytic bacteria from both cotyledons and embryonic axes, hence indicating that abundant and fast-growing core taxa, such as *Arthrobacter*, *P. agglomerans*, *Bacillus*, and Staphylococcus, displayed copiotrophy regardless of which seed compartment they were isolated from (Fig. 3 and 5A). Metabolic pathways interrupted during the latest phases of seed maturation drying are the first to be resumed with germination (23, 24), suggesting that copiotrophic bacterial taxa able to multiply during late maturation, including endophytes forming endospores and cysts (e.g., *Bacillus*) (28, 29), may have competitive advantages during germination. Following imbibition, endospores in embryonic tissues and proliferating bacterial cells between the seed coat and the root apical meristem have been detected in *Miscanthus* seeds (16). *Bacillus* and *P. agglomerans*, which are typical endospore-forming and niche colonization-versatile taxa (74, 75), accordingly appeared as the most abundant endophytes in both embryonic axes and cotyledons.

Satellite endophytic taxa were mostly cultured and isolated from the embryonic axis only, with *Curtobacterium* being among the few exceptions shared between the two seed compartments. In soybean and maize, isolates of this genus are found both in the seed endosphere and phyllosphere, and seed-mediated inheritance was proposed earlier by Dunleavy (76). Endophytes of the additional satellite genera *Brevundimonas*, *Methylobacterium*, *Microbacterium*, and *Rathayibacter* have been isolated from the seed endospheres of various crops and wild species (28, 36, 77–82). Different routes of endophyte vertical transmission from the parental plant to its progeny seeds include the so-called “internal pathway,” via xylem translocation, and the “floral pathway,” relying on the flower stigma as the entry point (5, 8, 19, 26, 83, 84). In terms of horizontal transmission and persistence in the plant, colonization of the embryonic axis has the advantage that this seed compartment will develop into a new plant, while cotyledons are more ephemeral. However, other studies on dry melon seeds and wheat seeds that had undergone imbibition detected higher abundance and diversity of endophytic bacteria in storage tissues and inner surfaces of seed coats rather than embryos (39, 85).

In summary, excising the embryonic axis from germinating seeds revealed broader endophytic richness and enabled isolation of poorly represented bacterial taxa, compared to the cotyledons, a second niche of the seed endosphere. Therefore, we suggest that trophic conditions of the embryonic axis promote endophyte diversity during soybean seed germination.

### Seed parental environment altered the diversity of culturable endophytic bacteria.

Environmental conditions and agricultural practices can influence seed endophytic communities, as revealed by metabarcoding analyses (43). Organic and conventional fertilization regimens, for instance, can modify the diversity of endophytic bacteria (86). ‘Bio-Amandine’ seeds, which belonged to the only lot produced following organic agricultural practices, possessed maximal strain diversity, hosted some satellite taxa, and also exhibited higher CFU numbers on 10% (wt/wt) TSA, relative to the other seed lots (Fig. 4 and 5B). While organic farming practice seems to curtail human-pathogenic bacterial genera, manure amendment and field proximity to livestock areas are sources of such bacteria, including Staphylococcus (58, 87, 88), which is already listed among seed endophytes (20, 27, 80, 81). Staphylococcus was abundantly isolated only from seeds of ‘Abelina’ and ‘Cordoba’ (Fig. 4), which were obtained from plants grown with conventional field fertilization, but not from ‘Bio-Amandine’, thus suggesting that Staphylococcus occurrence was not related to organic fertilization.

Remarkably, *Arthrobacter* and *Bacillus* appeared as core endophytic genera isolated from seeds of all lots, while endophytes of almost all other genera, excluding Staphylococcus, were seed lot specific (Fig. 4C) and included satellite genera and unique strains of the dominant and versatile taxa *P. agglomerans* and *Arthrobacter*, which commonly occur in soil (75, 89). Indeed, a prevalent fraction of seed endophytes appears to originate from the soil (84, 90, 91). Finally, in agreement with amplicon sequencing analyses (43, 92), and given that each seed lot was harvested from a distinct field (Fig. S2 in the supplemental material), we infer that local environmental conditions and agronomical practice in the parental environment substantially contributed to the microbiota composition of soybean seeds.

### Native endophytic bacteria displayed contrasting functional traits in the interactions with seeds under salt stress.

Studies on the functional role played by native bacterial strains isolated from the seed endosphere on seed germination remain rare and often limited to a few strains (20, 21, 93–95). To exemplify the potential held by the 246 isolates discussed in the present study, 39 strains, including all identified core and satellite genera, were assessed for their impact on seed germination under salt stress.

In at least three of the four seed lots, seed cross-inoculation with individual strains of a few satellite genera either accelerated (*Curtobacterium*) or slowed (*Microbacterium* and *Rathayibacter*) germination (Fig. 6), thus indicating that they may have functional relevance for seed germination under suboptimal conditions. This result aligns with previous findings on host-associated microbial communities (70, 96, 97). Similarly, in all seed lots, unique strains of the core genera *Arthrobacter* and *Bacillus* revealed slightly pathogenic effects by decreasing seed viability and vigor (Fig. 6), indicative of dysbiosis of the seed holobiont.

To summarize, challenging seeds of distinct lots with unique strains of both core and satellite taxa assisted in identifying potential functional traits affecting seed germination and vigor under salt stress. In conclusion, optimizing and combining various culturing approaches tailored to the endosphere of germinating seeds enabled isolation of a few unique bacterial strains affecting host performance under suboptimal germination conditions. Designing tailored media can pave a way forward in understanding functional traits of culture-recalcitrant endophytic bacteria relevant to their interactions with plants. Novel agricultural technologies are desirable to meet the rising demand of agricultural yields in increasingly erratic climates, on top of degrading and salinizing soils. Modulating the microbiotas of seeds may provide one option for ameliorating seed germination and promoting crop growth under suboptimal conditions.

## MATERIALS AND METHODS

### Seed germination, excision of seed compartments, and isolation of endophytic bacteria.

Seeds of four soybean (Glycine max L.) cultivars (i.e., ‘Abelina’, ‘Amadea’, ‘Bio-Amandine’, and ‘Cordoba’) were provided by Saatbau Linz eGen (Leonding, Austria; https://www.saatbau.com/standort/saatbau-linz-egen/) after harvest at full maturity in the 2019 crop season. Plants of each cultivar were grown in different fields in Upper Austria (Fig. S2 in the supplemental material), following conventional farming practices, excluding ‘Bio-Amandine’ seeds, which were produced under an organic farming management regimen. After harvesting, seeds of each cultivar were pooled in separate lots with an average water content of 6.5 ± 0.3% standard error (s.e.) on an FW basis, determined after 5 days of lyophilization. Subsequent seed handling and processing were conducted only aseptically, while workers were wearing 70% ethanol-cleaned latex gloves.

Several physical and chemical seed surface sterilization protocols were trialed (refer to, e.g., reference 98), including hot-steam-based treatments (12, 99), and led to successful eradication of epiphytic microorganisms, although no or just scant endophytic colonies were obtained on conventional rich complex media. Therefore, to prevent contamination from epiphytes and isolate culturable endophytes only, seeds (4 pools of 30 seeds each) were first submerged in autoclaved ultrapure water (aUPW) for 5 min at room temperature (RT) to ease aseptic seed peeling (i.e., removal of seed coats from individual seeds). Thereafter, peeled seeds underwent imbibition with aUPW on three layers of sterilized filter paper (Whatman grade 1; GE Healthcare, Little Chalfont, United Kingdom), followed by germination under sterility. Seed germination was defined as elongation of the embryonic axis’ radicle by at least 2 mm at 20°C in constant dark. Prior to excising the seed compartments (i.e., cotyledons and embryonic axes), the kinetics of seed germination for each lot were tracked up to first radicle elongation. Subsequently, 80% of incubation time before first germination (T80) was calculated to define the time points of excision from peeled seeds. Between individual seeds, excision and collection of seed compartments were always conducted with scalpels and tweezer cleaned with 70% (vol/vol) ethanol and flame sterilized.

For isolation, ~15 to 20 embryonic axes were gently removed from the cotyledons and ground in 5-mL autoclaved Teflon capsules with 10-mm-diameter agate beads (Sartorius, Göttingen, Germany), using a bead mill at 3,000 rpm for 1 min (Mikro-Dismembrator S; B. Braun Biotech International, Melsungen, Germany). The resulting paste was transferred to 2-mL autoclaved Eppendorf tubes with a 7-mm-diameter agate bead and resuspended in 1.5 mL of 0.9% (wt/vol) NaCl using a Tissue-Lyser (Qiagen, Hilden, Germany) at 30 Hz for 30 s. Similarly, cotyledons (*n *= 5 from different seeds) were first ground with 10-mm agate beads in 5-mL autoclaved Teflon capsules using a bead mill at 3,000 rpm for 6 min, prior to transferring of the paste to 20-mL Teflon capsules and resuspending in 5 mL of 0.9% (wt/vol) NaCl, as described for the embryonic axes. Following resuspension of both ground seed compartments, 15-μL aliquots of the slurries were streaked in quadruplicate on various agar-based media and incubated at 28°C in the dark until all colonies reached a size of ~0.5 to 1 mm. Blanks for both seed compartments were processed as real samples to exclude potential contaminations from equipment and solutions. Seed germination, excision of seed compartments, and grinding were repeated independently twice for each cultivar, resulting in eight independent isolation rounds.

### Media design and preparation.

Soybean-based solid media (1% [wt/vol] agar) were designed to simulate the nutritional conditions of T80 seeds. Therefore, the following six media, providing different concentrations and types of organic nutrients and inorganic salts, were designed. The first was 10% (wt/wt) TSA, obtained from tryptic soy broth (TSB; Corning Inc., Manassas, VA, USA), a standard complex and rich nonselective medium containing the enzymatic digest of soybean meal (peptone; https://www.corning.com/catalog/cls/documents/formulations/CLS-CG-FM-034.pdf), and included as soybean-based standard medium for bacterial culturing. The second was M9 minimal salts medium (Formedium, Hunstanton, United Kingdom), containing only inorganic salts (100), supplemented with 21 μM glucose and 26 μM galactose, as representative monosaccharides whose concentrations typically increase during soybean seed germination (73, 101). The third was M9 minimal salts medium supplemented with 4.1 mM Gln and 0.8 mM Glu, as the most abundant amino acids found in soybean seeds (73, 102). Being heat sensitive components, monosaccharides and amino acids were added through sterile filtration after autoclaving (121°C and 20 min for all six media). The fourth and fifth media were M9 minimal salts medium containing 1.8 to 3.5% (wt/wt) T80 seeds of the same cultivar used for endophyte isolation or 1.8 to 3.5% (wt/wt) T80 seeds only. Importantly, nonpeeled T80 seeds of all cultivars were germinated in separate petri dishes alongside those reserved for excision and ground with a bead mill, as reported for the cotyledons, prior to autoclaving. The last medium was 20% (vol/wt) bio-organic soy milk (Joya, Soja pur, Hain Celestial; Mona Oberwart Produktion Naturprodukte GmbH, Austria) with an average composition of 9% (wt/vol) soybean seeds, 2.3% (wt/vol) lipids (of which 0.3% [wt/vol] was saturated fatty acids), 0.9% (wt/vol) sugars (of which 0.8% [wt/vol] was sucrose), 0.6% (wt/vol) fibers, 3.9% (wt/vol) proteins, and 0.03% (wt/vol) NaCl. Slurries obtained from the same pools of embryonic axes or cotyledons were streaked on all six soybean-based media in each round of isolation, while all types of media without streaked slurries were included as controls.

### Culturing and quantification of seed endophytes.

Following incubation at 28°C in the dark for at least 24 h (see Table S1 in the supplemental material for the incubation time for each isolate), individual colonies were selected from all tested media based on dissimilar appearance (e.g., shape, color, size, surface, margin, etc.) to target the highest diversity of culturable bacterial endophytes. Whenever multiple colonies that displayed analogous phenotypes appeared on the same plate of a certain type of medium, two or three colonies with that phenotype were chosen and designated with the same identification number and consecutive letters (Table S1 in the supplemental material). To achieve the isolation of pure cultures, all selected isolates were streaked three times on 10% (wt/vol) TSA and incubated at 28°C in the dark until loop inoculation in 10% (vol/vol) TSB, followed by overnight growth (28°C, 180 rpm) and conservation at −80°C as glycerol stocks.

To quantify the numbers of endophytic CFU across the various media and seed lots, aliquots (10 μL) of the slurries obtained after grinding the embryonic axes (4 pools of 10 to 15) and cotyledons (4 pools of 5 excised from different seeds) of peeled T80 seeds were 10-fold serially diluted in 0.9% (wt/vol) NaCl. For each biological replicate, nine technical replicates were included, and plates with 0.9% (wt/vol) NaCl served as blank controls. After incubation (28°C, dark) for 24 to 48 h, the number of CFU was visually counted.

### Molecular identification of seed endophytes. (i) DNA extraction.

Bacterial DNA was extracted from 10% (wt/vol) TSB overnight cultures of each individual endophytic isolate using an UltraClean microbial DNA isolation kit (MO BIO Laboratories, Inc., Qiagen, Carlsbad, CA, USA) and eluted in 60 μL of aUPW, following the manufacturer’s instructions. The quality of 5-μL DNA aliquots was visually inspected after gel electrophoresis (100 V, 40 min) on 1% (wt/vol) agarose in TBE (89 mM Tris, 89 mM borate, 2 mM EDTA; pH 8.00) buffer and staining with 0.005% (vol/vol) Midori Green (Bulldog Bio, Inc., Portsmouth, England). Furthermore, DNA concentrations and purities were quantified using a NanoDrop 2000c spectrophotometer (Thermo Fisher Scientific, Waltham, MA, USA).

### (ii) 16S rRNA gene amplification and sequencing.

The 16S rRNA gene was partially amplified using the forward and reverse primers 8f (5′-AGA GTT TGA TCC TGG CTC AG-3′) and 1520r (5′-AAG GAG GTG ATC CAG CCG CA-3′), respectively (103, 104). Extracted DNA (0.5 μL) was added to a reaction buffer (Gibco BRL; Web Scientific, Crewe, United Kingdom) and combined with 2 mM MgCl_2_, 200 μM of each deoxynucleoside triphosphate (dNTP), 0.2 μM of both primers, and 2 U *Taq* polymerase (Gibco BRL; Web Scientific, Crewe, UK) in 50-μL PCR mixtures. PCR thermocycling started with an initial denaturing step of 5 min at 95°C, followed by 30 cycles consisting of 30 s at 95°C, 1 min at 54°C, and 2 min at 72°C, and completed by a final extension step of 10 min at 72°C. Amplicons were processed with a QIAquick PCR purification kit (Qiagen, Hilden, Germany), and their quality was checked after gel electrophoresis, as reported for DNA, prior to Sanger partial sequencing conducted by LCG Genomics (Berlin, Germany) with the primers 8f and 926r (5′-CCG TCA ATT CCT TTG AGT TT-3′) (105).

Retrieved sequences were visualized and inspected with the sequence alignment editor package BioEdit (Ibis Biosciences, Carlsbad, CA, USA), which allowed quality filtering and trimming. Finally, all sequences were assigned to bacterial genera using the suite tools Classifier (with 16S rRNA training set 18) and Seqmatch (with a confidence threshold of 85%) of the Ribosome Database Project (RDP; http://rdp.cme.msu.edu/index.jsp) (106) Taxonomy Platform (November 2020). Genus identification of endophytic isolates was further validated with the Nucleotide Basic Local Alignment Search tool (BLASTN) of the National Center for Biotechnology Information (NCBI), accessed in January and February 2021, considering the first 10 hits and excluding sequences from uncultured/environmental samples. Within the same genus, 16S rRNA gene sequences were initially clustered using the free software MultAlin (http://multalin.toulouse.inra.fr/multalin/) (107).

### (iii) RFLP analysis.

RFLP analysis of the IGS positioned between the 16S rRNA and the 23S rRNA genes was performed to discriminate closely related isolates (i.e., ribotypes) within the same genus. First, the IGS region was amplified using the primers pHr (5′-TGC GGC TGG ATC ACC TCC TT-3′) and P23SR01 (5′-GGC TGC TTC TAA GCC AAC-3′) (108). After the PCR mixtures were assembled as described for partial 16S rRNA gene amplification, thermocycling included an initial denaturing step of 5 min at 95°C, followed by 30 cycles consisting of 30 s at 95°C, 30 s at 54°C, and 1.5 min at 72°C, completed by a final extension step of 10 min at 72°C. Subsequently, 5 μL of each amplicon was digested with the endonucleases AluI, HhaI, and HhaIII (Thermo Fisher Scientific, Waltham, MA, USA) individually and incubated for 3 h at 37°C. The RFLP patterns of the resulting DNA fragments were visualized and assessed after agarose gel electrophoresis (2.5% [wt/vol], 80 V, 3 h) and staining with 0.005% (vol/vol) Midori Green.

### (iv) *gyrB* amplification and sequencing.

Isolates that were assigned to the Enterobacteriaceae based on partial 16S rRNA gene sequencing were further discerned at the strain level by partially amplifying the *gyrB* gene with a mixture of universal degenerate primers: UP1 (5′-GAA GTC ATC ATG ACC GTT CTG CAY GCN GGN GGN AAR TTY GA-3′), UP1G (5′-GAA GTC ATC ATG ACC GTT CTG CAY GCN GGN GGN AAR TTY GG-3′), UP2r (5′-AGC AGG GTA CGG ATG TGC GAG CCR TCN ACR TCN GCR TCN GTC AT-3′), and UP2Ar (5′-AGC AGG GTA CGG ATG TGC GAG CCR TCN ACR TCN GCR TCN GYC AT-3′) (109). Template DNA (1 μL) was added to a reaction buffer containing 2.5 mM MgCl_2_, 200 μM of dNTPs, 0.15 μM of each primer, and 4 U FIREPol DNA polymerase (Solis BioDyne, Tartu, Estonia) in 50-μL PCR mixtures. Thermocycling conditions included 5 min at 95°C and 35 cycles consisting of 1 min at 95°C, 1 min at 58°C, and 2 min at 72°C, completed with a final elongation step of 2 min at 72°C. The ~1,200-bp amplicons were purified with a QIAquick PCR purification kit (Qiagen, Hilden, Germany), and their quality was inspected after gel electrophoresis (1% [wt/vol] agarose, 100 V, 60 min), before Sanger sequencing by LCG Genomics (Berlin, Germany), using the primers UP1S (5′-GAA GTC ATC ATG ACC GTT CTG CAY-3′) and UP2rS (5′-AGC AGG GTA CGG ATG TGC GAG CC-3′). Sequences were first assessed by inspecting the quality of the chromatograms and then filtered and trimmed with the sequence alignment editor software BioEdit (Ibis Biosciences, Carlsbad, CA, USA). Contigs generated from the sequences of the various isolates, were aligned and hierarchically clustered using the free software MultAlin. Additionally, to identify the closest related species to the *gyrB* clusters, sequences were subjected to BLAST searches by accessing the NCBI repository in January and February 2021, after excluding sequences from uncultured/environmental samples. In cases of multiple hits with equal sequence identity, those obtained from whole-genome sequencing were chosen.

### Seed inoculation with native endophytic bacteria and germination under salt stress.

A collection consisting of 39 strains assigned to all the genera identified in the endosphere of germinating seeds of the four seed lots was grown on 10% (wt/wt) TSA containing 300 mM NaCl. For each strain, ratios between CFU number on 10% (wt/wt) TSA and optical density at 600 nm were established. Single colonies of each strain were loop inoculated in 10% (wt/vol) TSB and grown for 24 to 48 h at 28°C and 180 rpm. Cells were pelleted by centrifugation (4,500 rpm, 5 min) to remove 10% (wt/vol) TSB and washed once with sterilized 0.9% (wt/vol) NaCl, in which they were finally resuspended after a second centrifugation step (4,500 rpm, 5 min).

Pools of 15 seeds of the four lots were washed and vigorously shaken in aUPW for 30 s to remove debris from their surface. After discarding aUPW, seeds were inoculated with 10^6^ CFU mL^−1^ of each individual strain suspension for 2 h at RT and 120 rpm, and seeds incubated in sterilized 0.9% (wt/vol) NaCl were included as controls. Thereafter, excess cell suspensions were discarded, and seeds (3 replicates of 15 seeds each for every strain and seed lot) underwent imbibition in sealed petri dishes on three layers of filter paper (Whatman grade 1; GE Healthcare, Little Chalfont, United Kingdom) saturated with sterilized 300 mM NaCl to impose moderate salt stress. Samples were incubated at 20°C in the dark, and total germination, defined as number of seeds with radicles protruding by at least 2 mm, was scored daily for 7 days, when all seeds had germinated or started decomposing. The speed of germination, an index of seed vigor, was calculated as time to 50% germination (22).

### Statistical analyses.

Data distribution was based on quantile-quantile plots inspection and Shapiro-Wilk tests, while homogeneity of the variances across groups was ascertained with Levene’s tests. As data were not normally distributed, multiple comparisons were assessed for significance (α = 0.05) through nonparametric Kruskal-Wallis rank variance analyses followed by the Bonferroni correction. Differences in CFU numbers between embryonic axes and cotyledons were revealed after running nonparametric Mann-Whitney *U* tests, which were also used to compare fold changes in total germination and time to 50% germination between control and inoculated seeds under salt stress. All statistical analyses were performed using the software package SPSS Statistics 25 (IBM, New York, NY, USA).

### Data availability.

The partial 16S rRNA gene sequences of all bacterial isolates are available through the NCBI GenBank database under accession numbers ON715122 to ON715367.
