# Supplementary material for: Tailored Media Are Key to Unlocking the Diversity of Endophytic Bacteria in Distinct Compartments of Germinating Seeds
Source: Microbiol Spectr. 2022 Jul 18;10(4):e00172-22. doi: 10.1128/spectrum.00172-22 (PMC9431621; doi:10.1128/spectrum.00172-22)
Supplement: Supplemental file 2 — Supplemental material. Download spectrum.00172-22-s0002.pdf, PDF file, 0.4 MB [file spectrum.00172-22-s0002.pdf]

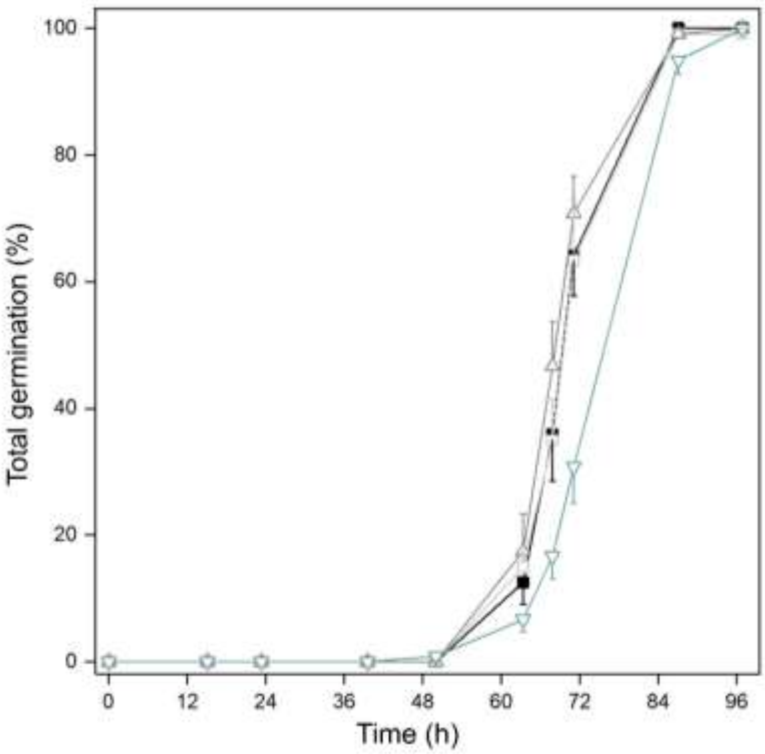

Supplemental Figure S1. Germination curves of soybean seeds of four lots at 20 °C in the dark. Each lot included seeds of a certain cultivar grown in a distinct field (refer to Supplementary Fig. S2) and is denoted by a unique symbol. Closed squares, 'Abelina'; open squares, 'Amadea'; closed triangles, 'Bio-Amandine'; open inverted triangles, 'Cordoba'. Prior to first radicle elongation [i.e., after 40 (for 'Cordoba') or 50 h (for the remaining seed lots) from the onset of imbibition], seeds were collected to isolate, molecularly identify, and count CFU numbers of bacterial endophytes. Data are means ± SE (*n* = 4 replicates of 30 seeds each).

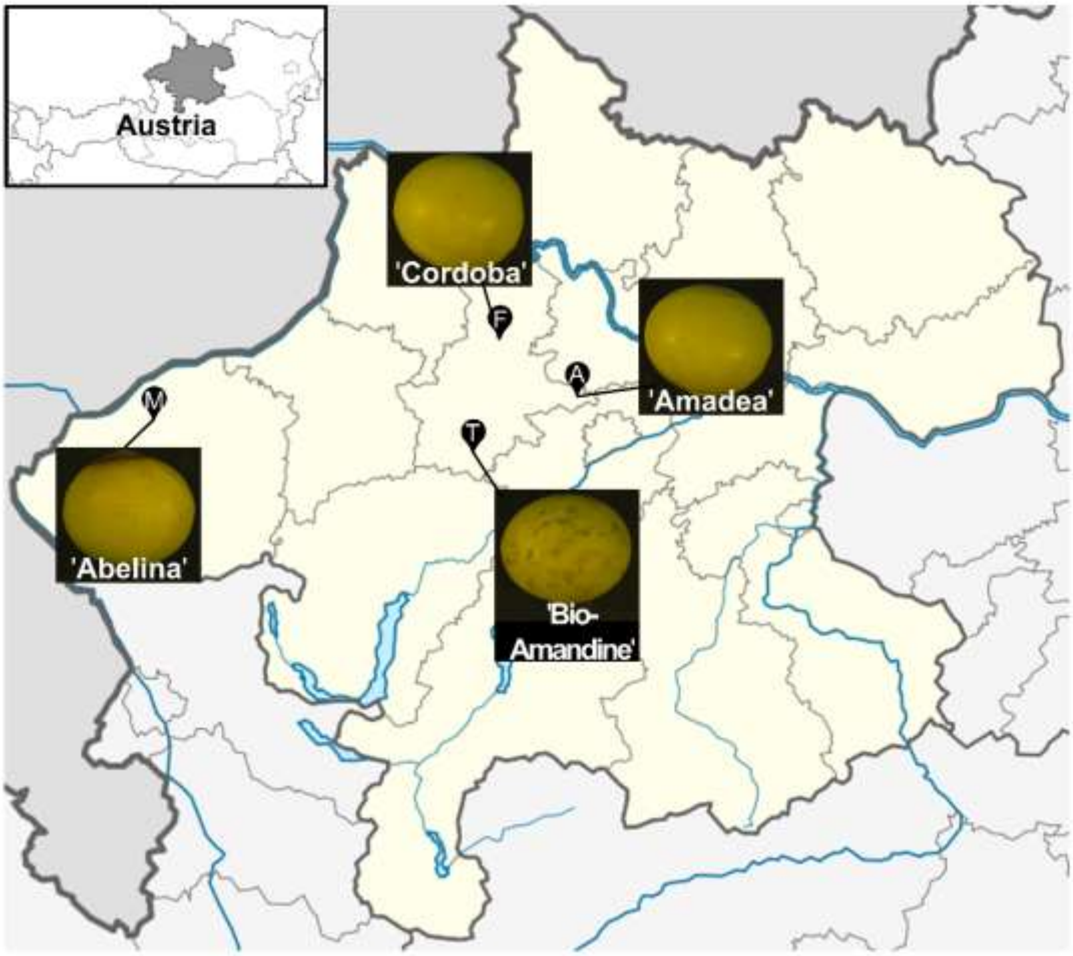

Supplemental Figure S2. Production sites of soybean seeds of different lots. Each seed lot was obtained from plants of a certain cultivar (i.e., 'Abelina', 'Amadea', 'Bio-Amandine', and 'Cordoba') grown in separate fields within the federal state of Upper Austria (see inset for its position within Austria). Letters indicate field locations in distinct areas of Upper Austria: A, Ansfelden; F, Feldkirchen; M, Moosbach; T, Thening.

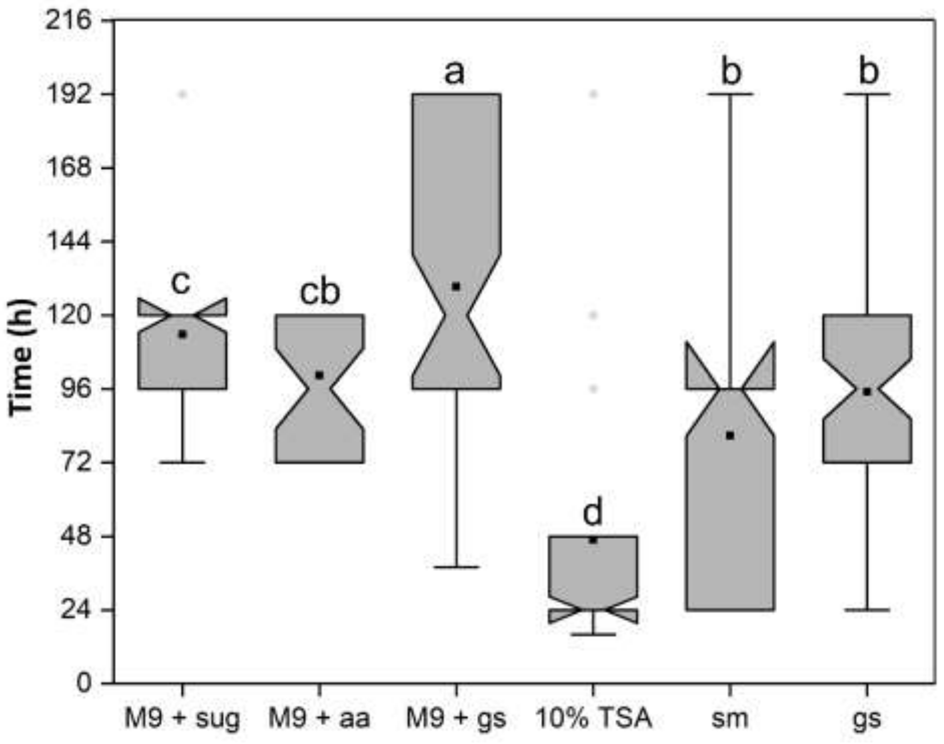

Supplemental Figure S3. Speed of growth of bacterial isolates from germinating soybean seeds on various solid (1% (w/w) agar) media. The time required to obtain colonies with a diameter of ~0.5-1 mm was used to estimate the speed of growth. M9 + sug: M9 minimal salts medium supplemented with 21 µM glucose and 26 µM galactose; M9 + aa: M9 minimal salts medium supplemented with 4.1 mM glutamine and 0.8 mM glutamate; M9 + gs: M9 minimal salts medium containing 1.8-3.5% (w/w) germinating and ground seeds; TSA: 10% (w/w) tryptic soy agar; sm: 20% (v/w) commercial soy milk; gs: 1.8-3.5% (w/w) germinating and ground seeds with agar only. Data are shown as notched box plots, with width proportional to the total number of isolates cultured on a certain medium. Closed squares and circles indicate means and outliers, respectively. Different letters denote statistically significant differences (non-parametric Kruskal Wallis test by ranks followed by the Bonferroni correction for multiple tests, $P < 0.05$).
